# Complete revascularization in elderly patients with myocardial infarction—a safe, effective, and necessary strategy?

**DOI:** 10.1016/j.xcrm.2023.101284

**Published:** 2023-11-21

**Authors:** Ying X. Gue, Gregory Y.H. Lip

**Affiliations:** 1 Liverpool Centre for Cardiovascular Science at University of Liverpool, Liverpool John Moores University and Liverpool Heart & Chest Hospital, Liverpool, UK; 2 Danish Center for Health Services Research, Department of Clinical Medicine, Aalborg University, Aalborg, Denmark

## Abstract

The FIRE trial compared culprit-only revascularization to physiology-guided complete revascularization strategy in elderly patients presenting with myocardial infarction. The study has shown that it is a safe approach and may confer additional prognostic benefit in patients with NSTEMI.

## Main text

With an aging population, the population presenting to medical facilities with acute coronary syndromes (ACSs) is also gradually getting older. Not only is age a strong predictor of adverse events in patients presenting with ACSs, but the presence of older age is also predictive of lower use of cardiac catheterization, which may contribute to poorer outcomes.^1^ The lack of inclusion and under-representation of older adults in randomized controlled clinical trials makes for an evidence-free zone for the management of this complex and high-risk cohort of patients. The Functional Assessment in Elderly MI Patients with Multivessel Disease (FIRE) trial^2^ set out to answer an important question in an increasingly common clinical scenario that is under-represented in clinical trials: does complete revascularization in elderly patients presenting with ACSs confer the same benefits as their younger counterparts?

In this multicenter, open-label randomized controlled trial, 1,445 patients aged at least 75 years old admitted to hospital with ACSs with successful percutaneous coronary intervention (PCI) to a culprit lesion and had residual disease in a non-culprit vessel were randomized to receive either physiology-guided complete revascularization or culprit-only revascularization. Of the 720 patients randomized to complete revascularization, 361 (50.1%) patients had revascularization in at least one non-culprit vessel. Results have shown a 27% reduction in composite of death, myocardial infarction, stroke, or ischemia-driven revascularization with the complete revascularization strategy. More importantly, there was a significant reduction of 36% in cardiovascular mortality, an outcome that was not evident in previous trials comparing the two different approaches with multi-vessel coronary artery disease.^3^

The FIRE trial establishes a few important points. First, a strategy for physiology-guided complete revascularization is safe in elderly patients presenting with multivessel coronary disease, with no difference in the occurrence of composite safety outcomes between the two groups. This is a highly important point as frailty, which commonly co-exists with advanced age, has been shown to be associated with higher mortality risk when presenting with myocardial infarction,^1^ and even though PCI can confer a survival benefit in this population,^4^^,^^5^ age and frailty could play a role in deciding the management strategy. Although procedure-related complications were not reported, the FIRE trial has taken this one step further when compared with previous studies^6^ to show that complete revascularization with PCI in the context of ACSs is safe in frail older patients in terms of peri-procedural complications (bleeding, stroke, and acute kidney injury). The occurrence of bleeding complications, with around half the cohort discharged on clopidogrel as the second antiplatelet, was not significantly different between the two groups. This is supportive of previous meta-analysis showing that the use of clopidogrel has been shown to be equally effective with reduced bleeding risk, conferring a greater net benefit.^7^

Second, complete revascularization could confer a reduction in cardiovascular mortality in a selected group of elderly patients with ACSs. The composite outcome was only significant in the non-ST-segment elevation myocardial infarction (NSTEMI) population as seen on the subgroup analysis. This is consistent with previous observational data on the benefits on long-term mortality following NSTEMI.^8^ Nonetheless, patients with NSTEMI are possibly more complex; hence, the definition of culprit vessel may be more convoluted as opposed to STEMI,^9^ which could explain the disparity in outcomes between the two presentations.

The FIRE trial protocol provides a robust guide on identifying the culprit lesion in patients with NSTEMI, which includes simple measures, such as electrocardiography, echocardiography, and angiography, to more complex invasive tests, such as intravascular ultrasonography, optical coherence tomography, and non-invasive imaging modalities like cardiac magnetic resonance imaging and speckle tracking. However, it is unclear how frequently these second-line investigations are utilized to define the culprit lesion within the 48-h window for randomization from PCI of culprit lesions within the median length of hospitalization of 6 days of the complete revascularization group. Therefore, the benefit of complete revascularization may potentially be in the context of a multi-culprit vessels coronary disease. On the flip side, as the culprit-only group did not have physiological assessment of their residual coronary disease, there could be a cohort with multi-culprit vessel coronary disease that did not achieve adequate revascularization of their presentation and hence confers a poorer prognosis.

Although age is an important factor in the decision toward the management strategy of ACSs, frailty would be more reflective of the condition of the patients. Analysis involving a frailty score, such as the clinical frailty score, would provide even more in-depth understanding of the risk and benefit of complete revascularization.

Overall, the FIRE trial has shown that complete revascularization is safe in elderly patients and confers a mortality benefit in the NSTEMI subgroup. This should be deliberated after an assessment of individual risk and benefit considering the presentation, comorbidities, and coronary anatomy. This approach will apply to patients that would be deemed suitable for invasive management of ACSs, which is another area up for debate that we hope the ongoing SENIOR-RITA trial^10^ will provide an answer for.
